# ERK1,2 Signalling Pathway along the Nephron and Its Role in Acid-base and Electrolytes Balance

**DOI:** 10.3390/ijms20174153

**Published:** 2019-08-25

**Authors:** Giovanna Capolongo, Yoko Suzumoto, Mariavittoria D’Acierno, Mariadelina Simeoni, Giovambattista Capasso, Miriam Zacchia

**Affiliations:** 1 Department of Translational Medical Sciences, University of Campania “Luigi Vanvitelli”, 80131 Naples, Italy; 2 Biogem Scarl, 83031 Ariano Irpino, Italy

**Keywords:** MAPK, ERK1,2, cell signaling, acid-base, electrolytes

## Abstract

Mitogen-activated protein kinases (MAPKs) are intracellular molecules regulating a wide range of cellular functions, including proliferation, differentiation, apoptosis, cytoskeleton remodeling and cytokine production. MAPK activity has been shown in normal kidney, and its over-activation has been demonstrated in several renal diseases. The extracellular signal-regulated protein kinases (ERK 1,2) signalling pathway is the first described MAPK signaling. Intensive investigations have demonstrated that it participates in the regulation of ureteric bud branching, a fundamental process in establishing final nephron number; in addition, it is also involved in the differentiation of the nephrogenic mesenchyme, indicating a key role in mammalian kidney embryonic development. In the present manuscript, we show that ERK1,2 signalling mediates several cellular functions also in mature kidney, describing its role along the nephron and demonstrating whether it contributes to the regulation of ion channels and transporters implicated in acid-base and electrolytes homeostasis.

## 1. Introduction

The MAPK cascade is a signal transduction common to several cell types, and it is involved in many biological processes, including cell proliferation, differentiation, development, and apoptosis [1]. 

The ERK1,2 signalling is the first identified MAPK signalling [2]. It is classically activated by extracellular stimuli as growth factors, cytokines, hormones, oxidative, and heat stress through several receptor types as tyrosine kinases (RTKs) and G protein-coupled receptors (GPCRs) [3]. The typical ERK1,2 cascade activation involves the recruitment of the guanine nucleotide exchange factor (SOS), which in turn activates the small G-protein, RAS [4]. The latter activates and recruits several downstream effectors, including isoforms of the serine/threonine kinase Raf, which is then de-phosphorylated at inhibitory sites and phosphorylated at activation sites. Following Raf activation, through a sequence of phosphorylations, MEK1 and 2 and subsequently ERK1,2 are activated. The latter has a number of effectors, including transcription factors, focal adhesion proteins and cytoplasmic proteins. Since several signalling pathways converge to ERK1,2 activation, the ability to localize activated ERK1,2 into cellular compartments is crucial to mediate specific cell responses. 

MAPK/ERK1,2 signalling has been shown to exert a significant role in renal morphogenesis and differentiation [5]. The activation of MAPK/ERK1,2 has been recently reported in both nephron progenitors (NPs) and developing kidney. Chemical MEK inhibition in rat embryos has been shown to affect nephrogenesis [6], and NPs-specific MAPK inactivation results in significant reduction of nephrons in newborn mouse pups [7].

MAPK signalling is activated by a wide range of stimuli also in mature kidney; these pathways have been shown to be critical in mediating cell response to different types of stress and injuries and to play a role in the pathophysiology of several diseases. Oxidant-induction of ERK1,2 signalling was demonstrated under ischemic injury, with ERK1,2 inhibition showing a favorable effect on the ischemia/reperfusion renal damage [8]. In addition, ERK1,2 activation has been described in polycystic kidney disease, some glomerulonephritis, diabetic nephropathy and unilateral ureteral obstruction, suggesting a role of this signalling in several renal diseases [9]. Interestingly, it has been shown also to participate in the regulation of the function of several channels and transporters along the nephron. The present manuscript describes the main evidence demonstrating the role of ERK1,2 signalling pathway along the renal tubule, with respect to acid-base and electrolytes balance. 

## 2. The Role of ERK1,2 Signalling along the Proximal Tubule (PT)

The PT has a key role in acid base, water, electrolytes and nutrients balance, by reabsorbing 60–70% of filtered NaCl, the bulk of filtered bicarbonate and almost all nutrients [10]. It is also the site of active solute secretion and hormone production, and it is involved in several metabolic functions of the kidney, as gluconeogenesis [11].

The PT contributes to the maintenance of acid-base homeostasis through multiple tasks: (1) by reclaiming almost all filtered bicarbonate (HCO_3_^−^); (2) by secreting protons (H^+^); (3) by synthetizing the major urinary buffer (NH_3_/NH_4_^+^) and new molecules of HCO_3_^−^ through glutamine metabolism and (4) by reabsorbing filtered citrate, which equals alkali reclamation [10]. Bicarbonate reabsorption and H^+^ secretion are fulfilled by a synergistic mechanism. H^+^ ions are mainly secreted into the lumen through the electroneutral Na^+^/H^+^ antiporter NHE3, expressed on the apical membrane. Secreted H^+^ combines with filtered HCO_3_^−^ into the luminal fluid, a reaction that is catalyzed by the luminal carbonic anhydrase (Ca IV), generating carbonic dioxide (CO_2_), which diffuses through the membrane into the cells, reconstituting H^+^ and HCO_3_^−^ [10]. Renal catabolism of glutamine is highly regulated by the acid base status and is the main determinant of net acid excretion (through increased ammonia synthesis) and of the generation of new HCO_3_^−^ molecules [12]. Increased urinary citrate reabsorption is another adaptive response to metabolic acidosis, as citrate reabsorption equals alkali reclamation. Increased activity of the luminal Na^+^-citrate cotransporter, NaDC1, is part of the adaptive response to an acid load [13], inducing a reduction of urinary citrate excretion [14].

**Figure 1:**
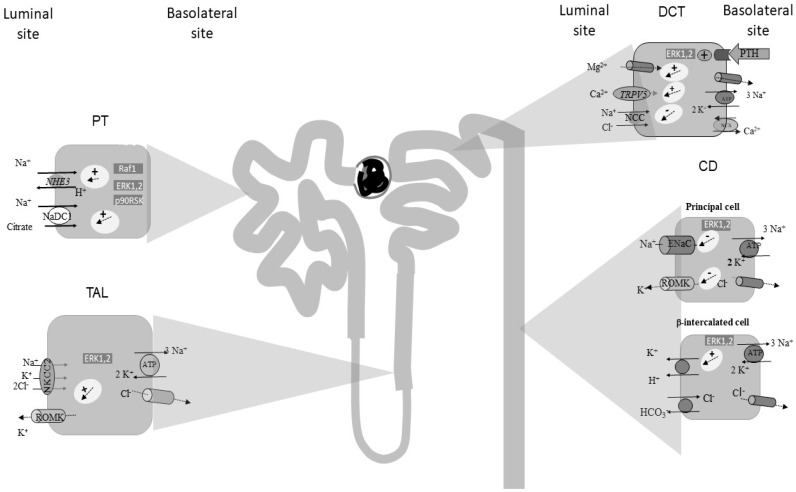
Schematic representation of ERK1,2 signalling regulation of channels and transporters along the nephron. Abbreviation: PT, proximal tubule; TAL, thick ascending limb of Henle’s loop; DCT, distal convolute tubule; CD, collecting duct.

The ERK1,2 signalling pathway has been demonstrated to be a key element-mediating adaptive response to decreased intracellular pH along the PT. Tsuganezawa et al. demonstrated that media acidification activates ERK1,2 in Opussum kidney cell line (OKP), a cellular model of PT [15]. In their study, ERK1 and 2 were immunoprecipitated from OKP cells and their activity measured by immune complex kinase assay using myelin basic protein as the kinase substrate. ERK activity picked up 5 to 15 minutes after media acidification. The effect was specific for PT cells, as media acidification had no effect on ERK1,2 activity in fibroblasts. In consistency with in vitro studies, ERK1,2 activity was shown to be increased also in mice renal cortex after 30 minutes from NH_4_Cl loading, indicating that also in vivo an acidic pH stimulated ERK1,2. Several evidences have suggested that the activation of ERK1,2 signalling by acid mediates the induction of several adaptive responses. MEK inhibition blocks ERK1,2 activation as well as acid stimulation of NHE3 activity, suggesting a role of ERK1,2 in the signalling pathway regulating acid-induced NHE3 activity. In addition, Li et al. showed that also angiotensin II-dependent stimulation of NHE3 is at least in part mediated by ERK1,2 signaling, in rodents [16], further supporting the role of MAPK signalling in NHE3 expression and activity. Further studies demonstrated that ERK1,2 phosphorylation and activation occur rapidly after acid loading both in vitro and in vivo. In OKP cells, media acidification caused increased phospho/total ERK1,2 ratio, which picked up 3–5 min after acid loading [17]. Specifically 30 min after NH_4_Cl gavage increased, ERK1,2 phosphorylation was observed in mice renal cortex. Interestingly, in OKP cells, ERK1,2 phosphorylation by media acidification was paralleled by increased Raf1 phosphorylation at serine 338, a marker of its activation, and by 90 ribosomal S6 kinase (p90RSK) phosphorylation, with a similar time course, suggesting that media acidification activates the typical Raf1/MEK/ERK1,2/p90RSK axis. Either Raf1, MEK and p90RSK inhibition blocked acid-induced NaDC1 activity in OKP cells. Taken together, these studies suggest that ERK1,2 signalling is activated by media acidification and an acid loading in vitro and in vivo respectively, and that the signalling mediates several adaptive responses to acidosis, namely increased NHE3 and NaDC1 activities (Figure 1). 

## 3. The Role of ERK1,2 Signalling along the Thick Ascending Limb of Henle’s Loop (TAL)

The loop of Henle is a peculiar nephron site with a characteristic anatomic configuration and specific properties. In normal subjects, the TAL reabsorbs 25–30% of filtered NaCl and plays a crucial role in calcium (Ca^2+^) reabsorption and urine concentration [18,19,20].

These functions are realized through the coordinated activity of several transporters and channels. Filtered NaCl enters the cells thanks to the presence of the luminal Na^+^/K^+^/2Cl^−^ cotransporter (NKCC2), in parallel, K^+^ recycles into the lumen via the K^+^ channel (ROMK) [21]. On the basolateral membrane, Na^+^ ions are pumped through the Na^+^/K^+^-ATPase system and a conductive Cl^−^ exit is mediated by a Cl^–^ channel. NKCC2 and in particular ROMK generate the “driving force” for paracellular reabsorption of Na^+^, K^+^, Ca^2+,^ and Mg^2+^ [22,23,24]. The paracellular cations transport is regulated by the calcium-sensing receptor (CaSR) expressed on the basolateral membrane of TAL cells, which modulates Ca^2+^ absorption, inhibiting ROMK activity and influencing the tight junction (TJ) structure [25,26,27].

To date, ERK1,2 signalling pathway activation has been described in intact renal tubular cells of the TAL [28,29,30].

In 1995, Terada et al. [31] showed that Raf-1, MEK, MAPK, and S6-K are ubiquitously expressed in rat renal tubular cells at mRNA and protein levels. In addition, they demonstrated that epidermal growth factor (EGF) significantly increased MAPK cascades, and endothelin (ET)-1 and ET-3 slightly stimulated MAPK cascades in the medullary thick ascending limb (MTAL).

In response to hypertonic stress, renal medullary epithelial cells modify their gene expression through the activation of the MAPK cascade [32,33], and p38 MAPK pathway was shown to regulate the function of specific membrane transporters in response to hypertonicity [34].

Gallazzini et al. examined the mechanisms by which tonicity regulates ROMK gene transcription in rat MTAL and in cultured mouse TAL cells [35]. In MTAL fragments, hypertonicity created by increased NaCl concentration, enhanced both ROMK mRNA abundance and the tonicity-responsive enhancer binding protein (TonEBP) total abundance and nuclear localization. Similar results were reported in immortalized mouse-derived TAL cell culture. Authors assessed that the inhibition of p38 MAPK pathway by its specific inhibitor SB203580 and of ERK1,2 by PD98059 abolished NaCl-induced stimulation of TonEBP and ROMK.

The MTALs participate also in the regulation of acid-base balance through luminal HCO_3_^−^ absorption thanks to the activity of the apical membrane Na^+^/H^+^ exchanger [36,37]. The hyperosmotic inhibition of HCO_3_^−^ absorption in the MTAL is mediated by different signalling pathways [38] with no experimental evidence of a direct activation of p38 MAPK, ERK or JNK, but is potentially influenced by growth factors or other extracellular mediators [39,40].

Finally, the distal nephron and particularly the medullary TAL segment plays an important role in response to the ischemic or acute renal damage. It has been demonstrated that after toxic ischemic renal injury, TAL cells show an altered expression/activation of many genes [41,42]. In this setting, EGF, by binding its receptor EGF-R, enhances renal tubular regeneration and repair after ischemic acute injury via RAS/RAF/ERK1,2 pathway, accelerating the recovery of renal function in post- ischemic injury [43,44].

Recent studies analysed in vitro the effect of several growth factors on human renal thick ascending limb and distal convoluted cells (TALDC) proliferation, demonstrating a significant, dose- and time-dependent phosphorylation of ERK1/2 after stimulation with EGF [45]. In contrast with PT cells, no other growth factors (HGF, nor IGF-1 or bFGF) induced a significant MAPK cascade activation in MTAL [46,47].

## 4. The Role of ERK1,2 Signalling along the Distal Convolute Tubule (DCT)

The DCT plays a key role in NaCl reabsorption and Ca^2+^ and Mg^2+^ handling [48,49].

Here, the sodium-chloride cotransporter (NCC), localized at the apical membrane, is responsible for the reabsorption of 5–10% of filtered Na^+^ and plays a key role in blood-pressure regulation [50]. Independent studies have demonstrated that NCC (down)regulation is mediated by ERK1,2 signaling. Ko and coworkers demonstrated that ERK1,2 signalling mediates NCC regulation by phorbol esters (PE) or functional analogs [51]. In their experiments, PE treatment decreased NCC surface expression through the stimulation of Ras guanyl-releasing protein 1 (RasGRP1)/Raf/MEK1,2 signalling. Interestingly, PE-induced NCC suppression was PKC-independent [52], while PKC inhibitors were without any effect; ERK1,2 inhibition totally prevented NCC downregulation. Ko’s group showed that the ERK1,2 pathway was crucial for NCC ubiquitination in response to PE administration in a cell culture model. In turn, the inhibition of the RasGRP1/ERK1,2 pathway prevented PE-induced NCC ubiquitination [51]. This was the first evidence of a physiological regulation of NCC by diacylglycerol (DAG), given that phorbol esters are functional analogues of DAG.

Parathormone (PTH) and Vitamin D are known to control Ca^+2^ and phosphate (PO_4_^−2^) homeostasis through direct actions on the PT and the DCT [53,54]. Ko and coworkers showed that PTH inhibits NCC activity via the Ras/ERK1,2 pathway [53]. Additional studies demonstrated that PTH induces ERK2 phosphorylation in both PT and DCT cells, but ERK1,2 inhibition blocks the enhanced PTH-dependent Ca^+2^ entry in mDCT only [55].

Both cell surface and total NCC expression are inhibited by the serine/threonine with no lysine kinase WNK4, via ERK1,2 signalling. Hypertonicity as well as EGF stimulated ERK1,2 phosphorylation in mDCT cells; these responses were totally blocked in the presence of mutations of WNK4, resulting in pseudo-aldosteronism type 2(PHA II), a rare form of hypertension associated with hyperkalemia [56,57]. NCC abundance is sensitive to dietary salt intake, and it is under hormonal control [58,59,60]. Lai et al. showed that high salt (HS) intake reduces NCC mRNA and protein abundance, enhancing ERK1,2 phosphorylation, while a low-salt (LS) diet produces the opposite effect, demonstrating that ERK1,2 mediates salt-dependent regulation of NCC [60]. Furthermore, this study showed that NCC modulation by salt intake is mediated by aldosterone via the WNK4-ERK1,2 mediated pathway. Feng et al. demonstrated that DUPS6, a MAP phosphatase-mediating ERK1,2, de-phosphorylation, is implicated in NCC modulation [61]. Besides ERK1,2 [53,61,62,63,64], also SPAK/OSR1 signalling plays a fundamental role in NCC regulation [65]. In SPAK KO mice, aldosterone resulted still able to regulate NCC abundance by controlling its ubiquitination via DUSP6-mediated ERK1,2 pathway, demonstrating that ERK1,2 and SPAK/OSR are independent pathways [61].

In the DCT, Ca^2+^ entry is mediated by the transient receptor potential vanilloid 5 (TRPV5), expressed across the apical membrane, while it is extruded by the basolateral Na^+^/Ca^2+^ exchanger (NCX1) [66]. Sneddon and coworkers observed that the activation of MAPK signalling by PTH is crucial for Ca^2+^ transport in DCT cells but is dispensable for the release of Ca^2+^ in the PT [53].

The DCT absorbs 10–15% of the filtered Mg^2+^ and plays a pivotal role in renal Mg^2+^ handling [67]. Cyclosporin A and FK506 are potent immunosuppressants and are known to exert several side effects, including hypomagnesemia [68]. Kim et al. examined the mechanisms underlying ciclosporin and FK506-induced hypomagnesemia. In this study, these medications inhibited PTH-dependent stimulation of Mg^2+^ uptake in mDCT cells, by inhibiting the ERK1,2 signalling pathway [69].

TRPM6 is a cation channel which transports mainly Mg^2+^, but also Ca^2+^ [70], exclusively expressed at the apical membrane of DCT cells. EGF has been demonstrated to regulate TRPM6 at the transcriptional level through the MEK/ERK/AP-1 pathway [71]. Indeed, patients with isolated autosomal recessive hypomagnesaemia, a genetic disorder defined by the mutation of EGF, exhibit increased urinary Mg^2+^ and decreased serum Mg^2+^ levels. In this inherited disease, the altered EGF leads to decreased activity and membrane expression of TRPM6, resulting in a decrease of Mg^2+^ reabsorption in the distal convoluted tubule [70].

## 5. The Role of ERK1,2 Signalling along the Collecting Duct (CD)

The CD consists of two types of cells: Principal cells (PCs) and α-β- intercalated cells (ICs). PCs mediate Na^+^ absorption through the amiloride sensitive-Epithelial Sodium Channel (ENaC) expressed at the apical membrane and regulated by aldosterone plasma levels [72]. Na^+^ absorption via ENaC is coupled to K^+^ secretion through the renal outer medullary potassium channel (ROMK) due to lumen electro-negativity [73,74]. ICs are principally specialized in the maintenance of acid-base balance, although an increasing number of evidences showed the involvement of ICs also in Na^+^ absorption [72]. α-ICs are responsible for acid secretion through the apical H^+^-ATPase and the basolateral Cl^−^/HCO^−^_3_ exchanger (AE1). In β-ICs, H^+^-ATPase is expressed on the basolateral membrane, while the Cl^−^/HCO_3_^−^ exchanger (Pendrin) on the apical membrane.

Interestingly, chronic lithium (Li^+^) administration induces morphological alteration of CD and downregulation of AQP2 in rat kidney medulla. In this context, rat inner medullary collecting duct (IMCD) phospho-proteomic profile was examined after acute lithium (Li) administration [75]. Nine hours after Li^+^ exposure, increased phosphorylation in activation sites of ERK1,2 and p38 were observed. Since pretreatment with the MAPK inhibitor abolished Li^+^-induced upregulation of pSer261-AQP2, ERK1,2 and p38, they have been suggested as early targets of Li^+^, possibly playing a role in the onset of Li^+^-induced polyuria [75]. After long term (15 days) Li^+^-treatment, ERK1,2 dysregulation was associated with a dysregulation of GSK3-beta [76], as it has been observed in other experimental models of polyuria, namely after ablating Dicer expression [77]. Whether there is an interaction between these two signalling pathways in regulating water reabsorption in the PCs remains to be investigated, but these data suggest a role of ERK1,2 in regulating urine concentration.

### 5.1. ERK1,2 Signalling in Principal Cells (PCs)

In the following paragraphs, the involvement of ERK 1,2 in the regulation of Na^+^ and K^+^ transport by PCs will be discussed.

#### 5.1.1. ENaC Regulation

The EGF exhibits a negative effect on amiloride-sensitive Na^+^ absorption [78]. Cotton’s group investigated the role of ERK1,2 in the regulation of amiloride-sensitive Na^+^ absorption through the Epidermal Growth Factor (EGF). In-vitro studies were conducted using primary and immortalized murine renal-collecting duct cells (mCT12); the cells were exposed to EGF, ATP, phorbol 12-myristate 13-acetate (PMA) or 2,5-di-tert-butyl-hydroquinone (DBHQ). In all conditions, increased ERK1,2 phosphorylation was observed, and ERK1,2 inhibition with PD98059 reduced EGF, ATP, and PMA-dependent inhibition of amiloride-sensitive Na^+^ absorption. ERK1,2-mediated inhibition of Na^+^ absorption was associated with phosphorylation of β- and γ-ENaC subunits, on the C-terminus [79]. Residues adjacent to PY motifs (β-Thr613, γ-Thr623) were proposed as phosphorylation sites and are believed to enhance the interaction between ENaC and the E3 ubiquitin-protein ligase, NEDD4, which targets proteins for ubiquitination [80]. However, in another cell line model expressing wild-type β-ENaC, ERK1,2 inhibitor completely reversed the EGF-induced inhibition of short circuit current (I_sc_) without affecting the surface expression of ENaC [81,82], complicating the intriguing mechanism of ENaC regulation by EGF. Recently, H-Ras was shown to be involved in EGF inhibition of ENaC [83]. Interestingly, in polycystic kidney disease (PKD), mis-location of the EGF receptor to the apical membrane of PCs and over-activation of its signal transduction, has been described. Accordingly, abnormal EGF-dependent regulation of ENaC activity has been described in an experimental model of PKD, and elevated ERK1,2 phosphorylation in cystic tubules was also implied in enhanced cellular proliferation, suggesting a possible link between ENaC de-regulation and renal cystogenesis [84].

#### 5.1.2. K^+^ Channels Regulation

Different types of K^+^ channels are expressed at the apical membrane of the CD, including ROMK, Ca^2+^-activated big-conductance K^+^ channels (BK) and K^+^ Two Pore Domain Channel Subfamily K Member 1 (KCNK1). ROMK and BK channels have been claimed as the main K^+^ channels [73]. Li and coworkers demonstrated that BK channel activity in both PCs and ICs is negatively regulated by p38 and ERK1,2. In fact, high K^+^ intake induced decreased ERK1,2 and p38 phosphorylation, resulting in increased BK channel activity [85]. Jin et al. [86] reported an inhibitory effect of Prostaglandin E2 (PGE2) on two apical K^+^ channels in CCD, ROMK-like small-conductance K^+^ channels (SK) and BK channels. PGE2, also known as dinoprostone, is a potent inflammatory mediator produced by cyclooxygenase 2 (COXII). In this study, PGE2 dramatically increased Protein kinase C (PKC)-dependent phosphorylation of ERK1,2 and p38. The effect of PGE2 was EP1 receptor-specific. Increased COXII expression and PGE2 production were induced by low-potassium intake. Even though the effect of K^+^-depletion on PGE2 production depends on the duration of diet and animal species, increased PGE2 level during K^+^-restriction might have a regulatory role on apical K^+^ channels in CCD through MAPK pathway and PKC [81]. In addition, superoxide anions have been demonstrated to mediate the effect of K-depletion on ROMK and renal K^+^ secretion through the phosphorylation of p38 and ERK, and the stimulation of protein tyrosine kinase (PTK) in CCD [87,88].

### 5.2. ERK1,2 Signalling in Intercalated Cells (ICs)

Laroche-Joubert and colleagues studied the signalling pathway-mediating isoproterenol-dependent stimulation of H^+^-K^+^-ATPase in rat CCD β-ICs, demonstrating the role of β-adrenergic receptors and Ras-Raf-1-ERK1,2 signalling [89]. Recently, a potential role of ICs on sterile inflammation in kidney has emerged [90]. In this study, Madin-Darby canine kidney cells originating from the renal collecting duct (MDCK C11) were used as a model system to characterize the purinergic receptor P2Y14 (GPR105). Proposed uridine diphosphate-(UDP-)glucose/P2Y14 signalling pathway for sterile inflammation in kidney initiates with the activation of P2Y14 expression by UDP-glucose, a damage-associated molecular pattern molecule (DAMP). P2Y14 expression was ICs-specific, and further increased ERK1,2 phosphorylation in MDCK C11 cells. The phosphorylation of ERK1,2 was shown to be responsible for the up-regulation of pro-inflammatory chemokine, such as IL-8 and CCL-2 (MCP-1).

## 6. Clinical Implications

### 6.1. ERK 1,2 and Hypomagnaesemia

Magnesium is crucially involved in several vital biological processes such as the regulation of enzymes and ion channels activity and the stabilization of ATP, ADP, RNA, and DNA. Severe magnesium deficiency can cause tetany, cardiac arrhythmia, and bone instability. As previously described, ERK 1,2 inhibition may lead to hypomagnesemia [68,69,70,71]. The chronic administration of cyclosporine or tacrolimus is associated with hypomagnesemia. These drugs, in fact, inhibit PTH-dependent magnesium uptake in the mDCT cells through the 1,2 ERK signalling pathway blockade. Additionally, several evidences deriving from clinical studies suggest that inhibiting ERK1,2 signalling results in hypomagnaesemia. The epidermal growth factor receptor (EGFR) is known to be activated by several ligands; upon its activation, it recruits more signalling intermediates, including ERK1,2. The use of anti-EGFR antibodies has been shown to cause hypomagnesemia. B-Raf and Mek inhibitors have been approved for clinical use in recent years. Hypomagnesemia and QT prolongation are described as non-rare side effects of the B-RAF inhibitor, vemurafenib.

In conclusion, we propose that in patients treated with calcineurin inhibitors (transplant recipients or glomerulopathies patients, etc.) and with drugs interfering with ERK1,2 signalling, serum Mg^2+^ level should be regularly monitored to prevent potentially fatal complications. More studies are expected to address this relevant focus.

### 6.2. ERK 1,2 Implications in Proteinuric Nephropathies and Hyperkalemia

It has been widely reported that ERK 1,2 activation is an important pathway sustaining podocyte injury in several proteinuric nephropathies. In fact, MAPK/ERK signalling pathway has been found to be activated in the mesangium of proteinuric patients [91]. In particular, this activation promotes a pro-inflammatory and pro-fibrotic cascade involving an increase of transforming growth factor-β1 (TGF-β1); plasminogen activator inhibitor-1 (PAI-1); MCP-1; IL-8; fibronectin and collagen expression in damaged renal tissue, indicating a role in fibrosis and, possibly, kidney disease progression. Anti renin-angiotensin-aldosterone (RAAS) agents are corner-stones of the antiproteinuric therapy [92,93], however, classical anti-RAAS molecules (ACE-inhibitors and AT1 receptor blockers) induce a compensatory increase in renin plasmatic activity that triggers prorenin-renin-ERK1,2-MAPK cascade activation. Aliskiren is a direct renin inhibitor able to overcome the renin escape with additive anti-proteinuric effects due to ERK 1,2-MAPK blockade [94]. This effect is particularly evident in patients with IgA nephropathy [95]. However, a major problem with aliskiren use is the risk of hyperkalemia development, that seems to be more evident with aliskiren than with other anti-RAAS agents. It could be supposed that an additive contribution to potassium retention depends on the effect of ERK 1,2 inhibition on potassium transport in the CD. Indeed, this aspect should be deepened in future studies.

### 6.3. ERk1,2 Implication in Sodium Handling and Hypertension

Blood pressure maintenance is mostly dependent on sodium balance, thus alterations of renal sodium handling are a contributing mechanism into the pathogenesis of salt-sensitive hypertension. RAAS is known to be the most important promoter of this condition. Recent reports demonstrated that all prorenin, renin and angiotensin II, and aldosterone are able to induce ERK 1,2 phosphorylation, and this might potentiate tubular Na^+^ retention-related hypertensive mechanisms [96]. Moreover, ERK 1,2 has been reported to play a role in vascular dysfunction associated with mineralocorticoid hypertension [97]. However, in a study by Rossol-Haseroth K and coworkers, mineralocorticoid receptor antagonists resulted inefficacious in blocking rapid ERK activation by aldosterone [98]. Of note, Lannoy M et al. proposed the use of ERK 1,2 inhibitors to reduce vascular stiffness and blood pressure in atherosclerotic patients [99]. However, we have reported above several studies demonstrating that ERK1,2 activation mediates the inhibition of crucial Na^+^ transporters and channels, as NCC and ENaC. Whether hypertension, a common side effect of drugs inhibiting BRAF, may be the consequence of the loss of this inhibition, requires additional studies.

### 6.4. ERK 1,2 Implications in Cancer

The activation of the ERK1,2 signalling pathway is the result of several proliferative signals, however, it is not surprising that its overactivation has been found in many forms of cancer. MAPK pathway is estimated to be deregulated in almost 50% of human malignancies, activating mutation of BRAF in 5–10% of cancers, especially hairy cell leukemia, melanoma, colorectal cancer, papillary thyroid cancer, non-small cell lung cancer (NSCLC), and ovarian cancer [100]. Thus, targeting the ERK1,2 signalling pathway has emerged as a key element in clinical oncology. Given the broad expression of ERK1,2 signalling and its role in several physiological functions in almost all organs and tissues, it is clear that a number of side effects are expected. Selective inhibition of the MAPK signalling pathway has been approved in recent oncological care. BRAF and MEK inhibitors are currently used in advanced melanomas harbouring BRAF-activating mutations [101]. Direct ERK1,2 inhibitors are under investigation. Few molecules, including BVD-523 and GDC0994, have reached clinical trials recently. Given the fact that these medications are relatively new, it is possible that a number of side effects are still poorly defined; in fact, little is known on their putative effects on acid-base and electrolytes balance. Hypomagnesemia, hypokalemia and hypocalcemia have been described in patients under vemurafenib treatment. In addition, several immunosuppressants with different mechanisms of action have been shown to interfere with the ERK signalling pathway. It is the case of ciclosporin A and FK506, as described before, with relevant electrolytes imbalance due to ERK1,2-mediatet regulation of channels and transporters.

### 6.5. ERK 1,2 Implications in Acid-Base Imbalances

As described in the previous paragraphs, the ERK 1,2 signalling pathway is involved in acid-base regulation due to its influence on several tubular transporters. Specifically, it has been widely demonstrated in numerous in vitro and in vivo studies that the ERK 1,2 pathway is activated by intracellular acidosis and mediates compensatory responses [14,15,16,17]. Drugs inhibiting ERK 1,2 signalling are expected to reduce important adaptation responses to metabolic acidosis. Thus, acid-base status should be carefully evaluated in the course of treatment with ERK 1,2 inhibitors. Indeed, this focus deserves scientific attention in future studies.

## 7. Conclusions

ERK1,2 signalling pathway is known to be critical in embryonic kidney development. The present review shows that it mediates the transcriptional and post-transcriptional regulation of several nephron channels and transporters in mature organs. The pivotal role in kidney homeostasis is further demonstrated by the over-activation in several renal diseases. The clinical use of molecules targeting ERK1,2 signalling, especially in oncology, is expected to influence numerous vital nephron functions. In conclusion, a close monitoring of acid-base and electrolytes balance parameters, together with glomerular function markers, should be a routine part of clinical care in patients treated with drugs interfering with ERK1,2 signalling pathway.
